# Widely applicable, extended flow cytometric stem cell enumeration panel for quality control of advanced cellular products

**DOI:** 10.1038/s41598-022-22339-1

**Published:** 2022-10-26

**Authors:** Katy Haussmann, Mathias Streitz, Anna Takvorian, Jana Grund, Zemra Skenderi, Carola Tietze-Bürger, Kamran Movassaghi, Annette Künkele, Agnieszka Blum, Lars Bullinger

**Affiliations:** 1 Corporate Member of Freie Universität Berlin, Humboldt Universität Zu Berlin, and Berlin Institute of Health, Stem Cell Facility, Charité–Universitätsmedizin Berlin, 10353 Berlin, Germany; 2 Institute of Medical Immunology, Corporate Member of Freie Universität Berlin, Humboldt-Universität Zu Berlin, and Berlin Institute of Health, Charité – Universitätsmedizin Berlin, Augustenburger Platz 1, 13353 Berlin, Germany; 3 Department of Experimental Animal Facilities and Biorisk Management, Friedrich-Loeffler Institut, Greifswald-Insel Riems, Germany; 4 Department of Pediatric Oncology and Hematology, Corporate Member of Freie Universität Berlin, Humboldt Universiät Zu Berlin, and Berlin Institute of Health, Charité–Universitätsmedizin Berlin, 10353 Berlin, Germany; 5 German Cancer Consortium (DKTK), 10117 Berlin, Germany; 6 German Cancer Research Center (DKFZ), 69120 Heidelberg, Germany; 7 Berlin Institute of Health at Charité - Universitätsmedizin Berlin, Charitéplatz 1, 10117 Berlin, Germany; 8 Ardigen, 30-394 Kraków, Poland; 9 Department of Hematology, Oncology and Tumorimmunology, Corporate Member of Freie Universität Berlin, Humboldt Universität Zu Berlin, and Berlin Institute of Health, Charité – Universitätsmedizin Berlin, Berlin, Germany

**Keywords:** Immunology, Stem cells, Oncology

## Abstract

The most widely used quality control assay for CD34 + hematopoietic stem cell product characterization is the protocol established by the International Society of Hematotherapy and Graft Engineering (ISHAGE). While this protocol is still the gold standard for stem cell enumeration and viability assessment, it does not include T cell enumeration, which is nowadays mandatory for assaying standard allogeneic grafts and various advanced therapy medicinal products (ATMPs). In accordance, we have developed and extensively validated a new approach for a more comprehensive characterization of hematopoietic cellular products using a pre-formulated dried antibody format panel. In addition to the counting beads, the typical markers CD45 fluorescein isothiocyanate (FITC) and CD34 phycoerythrin (PE), as well as the viability dye 7-amino actinomycin D (7-AAD), our novel pre-formulated panel also contains CD3 Pacific Blue (PB) and CD19 allophycocyanin (APC) in the same tube, thereby allowing a combined calculation of leucocytes, stem cells, T and B cells. Showing high linearity, sensitivity and accuracy, our approach is easy to implement and enables a more in-depth characterization of the cellular product under release testing conditions. In addition, the dried pre-formulated antibody approach increases assay reliability compared to the standard antibody panel.

## Introduction

The success of stem cell transplantation depends on the collection and infusion of adequate numbers of viable CD34^+^ hematopoietic stem cells. CD34 enumeration by flow cytometry is a widely accepted method to count hematopoietic progenitors and stem cells. In 1996 the Stem Cell Enumeration Committee of the International Society of Hematotherapy and Graft Engineering (ISHAGE) published the guideline for CD34^+^ cell determination^1^ that remains the recognized standard for laboratories all over the world. Over the time, the protocol for stem cell enumeration has been optimized^1–5^. Following the original publication of the ISHAGE standards by Sutherland et al.^1^ Keeney et al*.* modified the protocol again introducing a single platform method with counting beads, two colors and the cell death marker 7-AAD^1,4^.

Since then, the ISHAGE protocol has become a recognized pharmaceutical guideline. However, the current advances in the development of cellular therapies enforce the further improvement of quality control (QC) assays. In fact, information about the residual CD3 count is important for products such as donor lymphocyte infusions, TCR α/β- and CD19-depleted haploidentical allogeneic stem cell grafts. In addition, the leucocyte and T cell counts are also relevant for advanced therapy medicinal products (ATMP) such as autologous mononuclear cells in extracorporeal photopheresis (ECP) or genetically engineered chimeric antigen receptor (CAR) T cells and CAR natural killer (NK) cells^6–9^. Furthermore, quantification of B cells gains on importance since there is a growing body of evidence indicating that B cell transfer can support the humoral immune response after hematopoietic stem cell transplantion^10,11^. Moreover, new generations of clinical flow cytometers expand the technical capabilities far beyond the detection of just three fluorescence parameters^12^.

In order to meet the emerging QC requirements, we have established an innovative expanded, highly standardized and easily applicable flow cytometric panel. Besides the enumeration of CD34 cell subset, this panel also includes quantification of CD3 and CD19 cells. Furthermore, the staining protocol was optimized using the DURAClone dry technology. This approach is based on a dry, pre-formulated, custom-made reagent panel that enables robust performance and a faster turn-around of the assay.

Here, we present the validation results for this simplified and standardized approach that enables not only CD34 quantification, but also the determination of CD3 and CD19 cells in peripheral blood, as well as in fresh and cryopreserved standard stem cell products. The implementation of our assay allows an optimized, lean and efficient QC for a wide range of cell therapeutics. Furthermore, this approach is flexible and can be further adapted to suit the specific needs for the characterization of novel cellular products.

## Results

The validation of our novel method was specified in a validation plan that defines the types of specimens to be investigated [whole blood (WB) and leukapheresis (LA)], the number of samples and replicates, as well as the acceptance criteria for internal laboratory range, linearity, sensitivity, accuracy and inter-operator analysis variation. This validation plan is summarized in Table 1. In general, the total number of samples does not include the CD34 isoclone control sample. Within the accuracy testing the new assay was compared with established reagent panels in liquid format.

**Table 1:** Summary of the validation design for the pre-formulated dried reagent panel (CD45 FITC, CD34 PE, 7-AAD, CD3 PB, CD19 APC as well as absolute counting beads) measured with the flow cytometer Navios 10/3 with different sample types of standard products.

Performance characteristic	Used sample types	Internal laboratory range	Linearity	Sensitivity	Accuracy	Inter-operator analysis
	Non-mobilized WB	×	–	–	–	×
	Mobilized WB	×	×	×	×	×
	Mobilized stem cell apheresis	×	×	–	×	×
	Mobilized stem cell apheresis after cryopreservation	×	–	–	×	–
	Donor lymphocytes	×	×	–	×	×
	Raji cell line	–	–	×	–	–
	Stem cell apheresis-CD34 selected	–	–	×	–	–
	Non transduced CD4/CD8 cells	–	–	×	–	–
	Reference blood	–	–	–	×	–
No. of samples with different cell concentrations		185	24	5	148	5
No. of replicates		1–3	2	10	1–3	1–2
Total no. of samples		295	48	50	324	8
Acceptance criteria		–	r^2^ ≥ 0.95	–	R^2^ ≥ 0.95 reference values pass CV ≤ 10%	CV ≤ 10%

### Establishment of the pre-formulated dried reagent panel: internal laboratory measurements reveal typical wide laboratory ranges

In the manufacturing of pharmaceuticals, QC with the analysis of active ingredients plays a central role, since products and product batches can only be released after product specifications have been determined and evaluated. The characterization of cellular products forces the respective laboratories to establish measurement ranges for the different parameters determined during QC testing. Because of the specific character of these products, they naturally differ from the reference values of the blood obtained from healthy donors. Therefore, we used the laboratory range to give an overview about the measuring range for CD45, CD34, CD3 and CD19 populations, respectively, and statistical values (minimum (min), maximum (max), median (median)) in standard cellular products and their in-process controls (n = 295) at the Charité Stem Cell Facility (CSCF). The obtained values reflected the wide ranges generally observed in the QC measurements (Table 2).

**Table 2:** Results of the Laboratory range: Several sample types of patients and donors (n = 295) were measured to confirm the laboratory range of frequencies (%), viable absolute values (cells/µL) and viability (%) for the parameter CD45, CD34, CD3 and CD19.

Parameter		Min	Max	Median
CD45	cells/µL	1700	783,840	79,420
	Viability %	37.33	100.00	98.75
CD34	%	0.00	11.48	0.32
	cells/µL	0	35,440	328
	Viability %	34.21	100.00	99.46
CD3	%	1.26	62.72	15.83
	cells/µL	252	200,040	6,024
	Viability %	85.14	100.00	99.03
CD19	%	0.00	17.22	1.75
	cells/µL	0	60,120	644
	Viability %	0.00	100.00	99.23

The new approach nicely reflected the expected difference between the specimens and cohorts (Supplemental Table S1). As expected, healthy donor derived samples showed higher absolute values for CD45 and CD34 as well as higher CD34 frequencies after granulocyte-colony-stimulating factor (G-CSF) mobilization than specimen from healthy donors without stimulation. Measurements of stimulated WB samples before apheresis (n = 15) showed the following results [CD45 cells/µL: 56,280 (28,860–84,620); CD34 cells/µL: 120 (32–280); CD34%: 0.19 (0.11–0.47)], and measurements in stimulated allogenic cellular products (n = 30) the following findings [CD45 cells/µL: 280,805 (116,695–691,240); CD34 cells/µL: 2020 (460–4800); CD34%: 0.72 (0.24–2.68)]. In comparison WB analysis of specimen from healthy donors without stimulation (n = 15) showed the following [CD45 cells/µL: 5770 (4080–8200); CD34 cells/µL: 2 (0–4); CD34%: 0.03 (0–0.08)], and the investigation of unstimulated donor lymphocytes (n = 20) these results [CD45 cells/µL: 78,315 (29,730–114,090); CD34 cells/µL: 57 (8–267); CD34%: 0.07 (0.01–0.24)]. In stimulated WB before LA the median CD45 absolute count was lower than after LA [CD45 cells/µL: 30,975 (1700–84,620) vs. CD45 cells/µL: 41,330 (16,990–85,200)], whereas the median CD34 absolute count and its frequency were higher [CD34 cells/µL: 112 (0–595); CD34%: 0.19 (0.00–5.61) vs. CD34 cells/µL: 37 (5–660); CD34%: 0.11 (0.02–1.75)]. Furthermore, the median values of the CD45, CD34 and CD3 absolute counts were lower in LA samples of mobilized patients than of healthy mobilized donors [CD45 cells/µL: 166,390 (64,490–783,840); CD34 cells/µL: 1285 (111–35,440); CD34%: 1.12 (0.13–11.48); CD3 cells/µL: 23,662 (2977–70,440); CD3%: 14.84 (3.63–43.29); CD19 cells/µL: 266 (0–6670); CD19%: 0.19 (0.00–3.03) vs. CD45 cells/µL: 280,805 (116,695–691,240); CD34 cells/µL: 2020 (460–4800); CD34%: 0.72 (0.24–2.68); CD3 cells/µL: 68,020 (41,190–200,040); CD3%: 25.77 (12.56–37.20); CD19 cells/µL: 17,980 (1207–60,120); CD19%: 6.51 (2.69–14.39)]. The median values of the viability parameter were close to 100% but the analysis showed low viability for CD45 (37%) and CD34 (34%) and the minimum viability of CD19 was zero. At the end, we defined our laboratory range based on the parameters measured in all different specimens used.

### The new assay linearity was confirmed in standard cellular products and in process controls

The linearity indicates the level of correspondence between the measured cell phenotypes and the expected concentrations of CD45, CD34, CD3 and CD19 cells in the sample stained with the pre-formulated dried reagent panel. The coefficient of determination (r^2^) is determined by the calculated expected value compared with the analysis value. A high coefficient of determination close to 1.0 indicates high linearity since the measured cell concentrations are then directly proportional to the calculated expected cell concentration in the sample.

**Figure 1:**
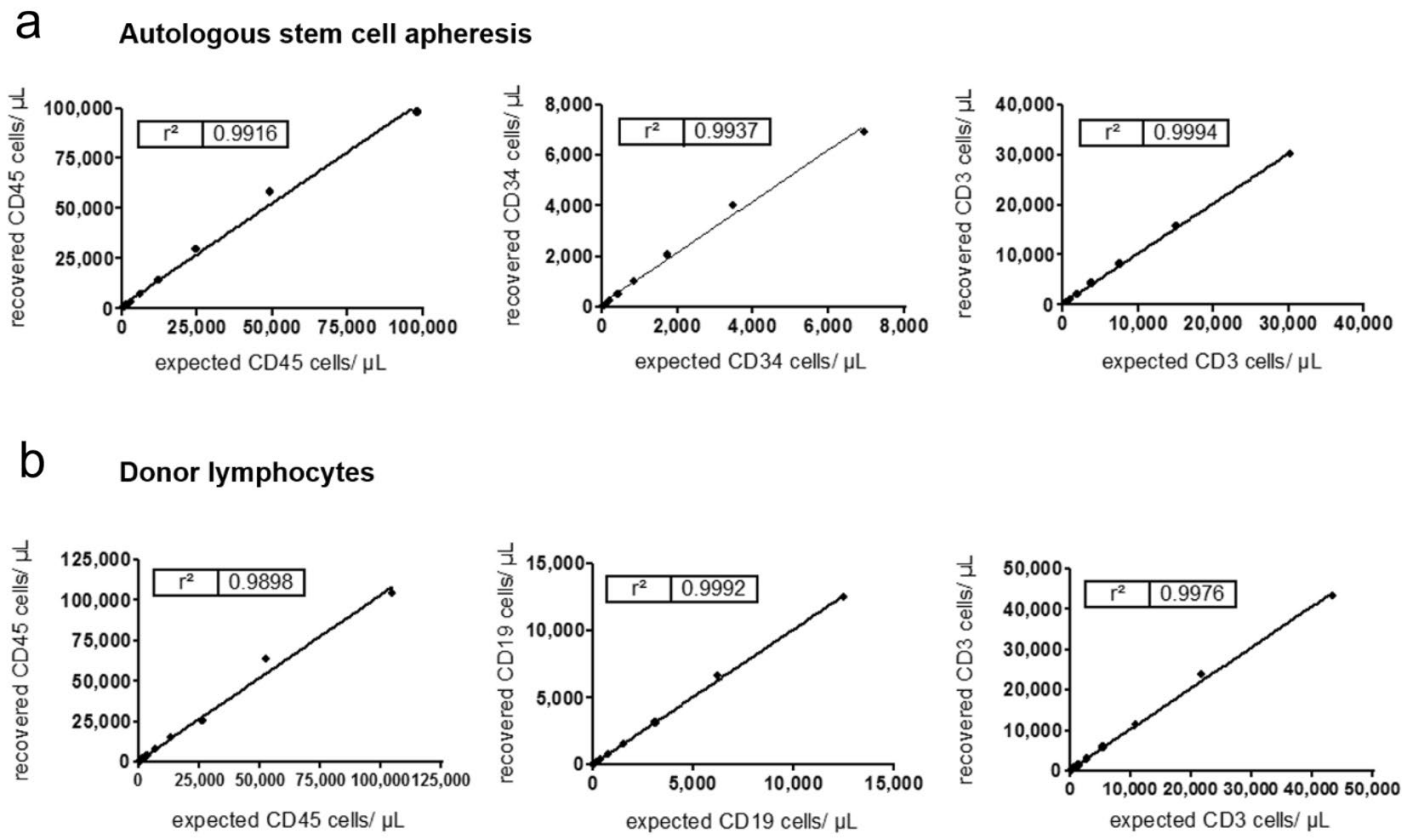
Linearity results of method validation with the pre-formulated dried reagent panel (CD45 FITC, CD34 PE, 7-AAD, CD3 PB, CD19 APC, counting beads) and standard products analyzed with the flow cytometer. Autologous stem cell apheresis (**a**) and donor lymphocytes (**b**) were serial diluted and the concentration means of recovered cells in cells/µL (A: CD45, CD34, CD3; B: CD45, CD3, CD19) were compared with the expected values by linear regression*.*

Fresh autologous stem cells and non-mobilized healthy donor lymphocytes were stained with the pre-formulated dried reagent panel for linearity testing (Fig. 1). In fresh autologous stem cells, linearity was confirmed for the CD45 cell concentration between 975 and 98,324 cells/µL (r^2^ = 0.9916), for CD34 between 61 and 6931 cells/µL (r^2^ = 0.9937) as well as for CD3 between 552 and 30,199 cells/µL (r^2^ = 0.9994) (Fig. 1a). In lymphocytes of healthy donors, the linearity was measured for CD45 cells between 410 and 104,596 cells/µL (r^2^ = 0.9898), for CD3 between 145 and 43,254 cells/µL (r^2^ = 0.9976) and for CD19 between 38 and 12,481 cells/µL (r^2^ = 0.9992) (Fig. 1b). Linearity was also compared in mobilized WB and in fresh autologous stem cells. Despite different CD34 levels, the r^2^ for mobilized WB with lower CD34 levels were comparable with the one seen in the fresh autologous stem cells with higher CD34 levels. The acceptance criteria of r^2^ ≥ 0.95 for linearity were always met across the tested samples and for all populations of interest. The linearity over all tested samples was therefore confirmed for the CD45 concentration between 761 and 98,324 cells/µL, for the CD34 concentration between 2 and 6931 cells/µL as well as for the CD3 concentration between 55 and 43,254 cells/µL and for the CD19 concentration between 12 and 12,481 cells/µL.

### Sensitivity measurement strategies result in different values for LOD and LOQ

The sensitivity of a measurement can be described using the following values: Limit of Blank (LOB), Limit of Detection (LOD) and Limit of Quantification (LOQ). To measure the sensitivity, two procedures were tested. The established approach includes LOD and LOQ determination whereas the novel approach additionally determines the LOB. With the help of low-level samples, the LOD and LOQ of the established approach can be determinate. Low-level samples of CD34 cells, CD3 cells and CD19 cells identified with the in the Charité Stem Cell Facility reference method by the FACS Calibur flow cytometer before the elaborate sensitivity measurements began. Reference samples without CD34 cells (WB), CD3 cells (Raji cell line) and CD19 cells (CD4/CD8 selected cells) were used for LOB determination. For every parameter CD34, CD3 and CD19 and sensitivity parameter 10 replicates analyzed and statistical evaluated. The assay with the pre-formulated dried panel in combination with the flow cytometer Navios showed a very low LOQ for both frequency levels and absolute counts (Table 3). Both measurement strategies showed comparable results for the LOQ and the LOD (Supplemental Tables S2 and S3). The LOQs of CD34 cells were similar and showed in both approaches a lower sensitivity level than the LOQs for CD3 and CD19 cells. Additionally, sensitivity analysis with the established approach (CD34: 2 cells/µL; 0.04%; CD3: 6 cells/µL; 0.40%; CD19: 5 cells/µL; 0.06%) was performed to confirm the validity of the new release testing assay.

**Table 3:** Sensitivity results summary for of the Limit of Detection (LOD) and Limit of Quantification (LOQ) measured as established approach for frequencies and absolute counts of the parameters CD34, CD3 and CD19.

Parameter		Sensitivity established approach		Sensitivity novel approach		
		LOD	LOQ	LOB	LOD	LOQ
CD34	%	0.01	0.04	0.01	0.02	0.03
	cells/µL	1	2	0	1	2
CD3	%	0.12	0.40	0.02	0.21	0.22
	cells/µL	2	6	0	3	4
CD19	%	0.02	0.06	0.00	0.03	0.04
	cells/µL	1	5	0	3	4

The novel approach of sensitivity measurements estimates additionally the Limit of Blank (LOB) by Armbruster et al.^13^.

### Measuring cells in one tube improves accuracy

**Figure 2:**
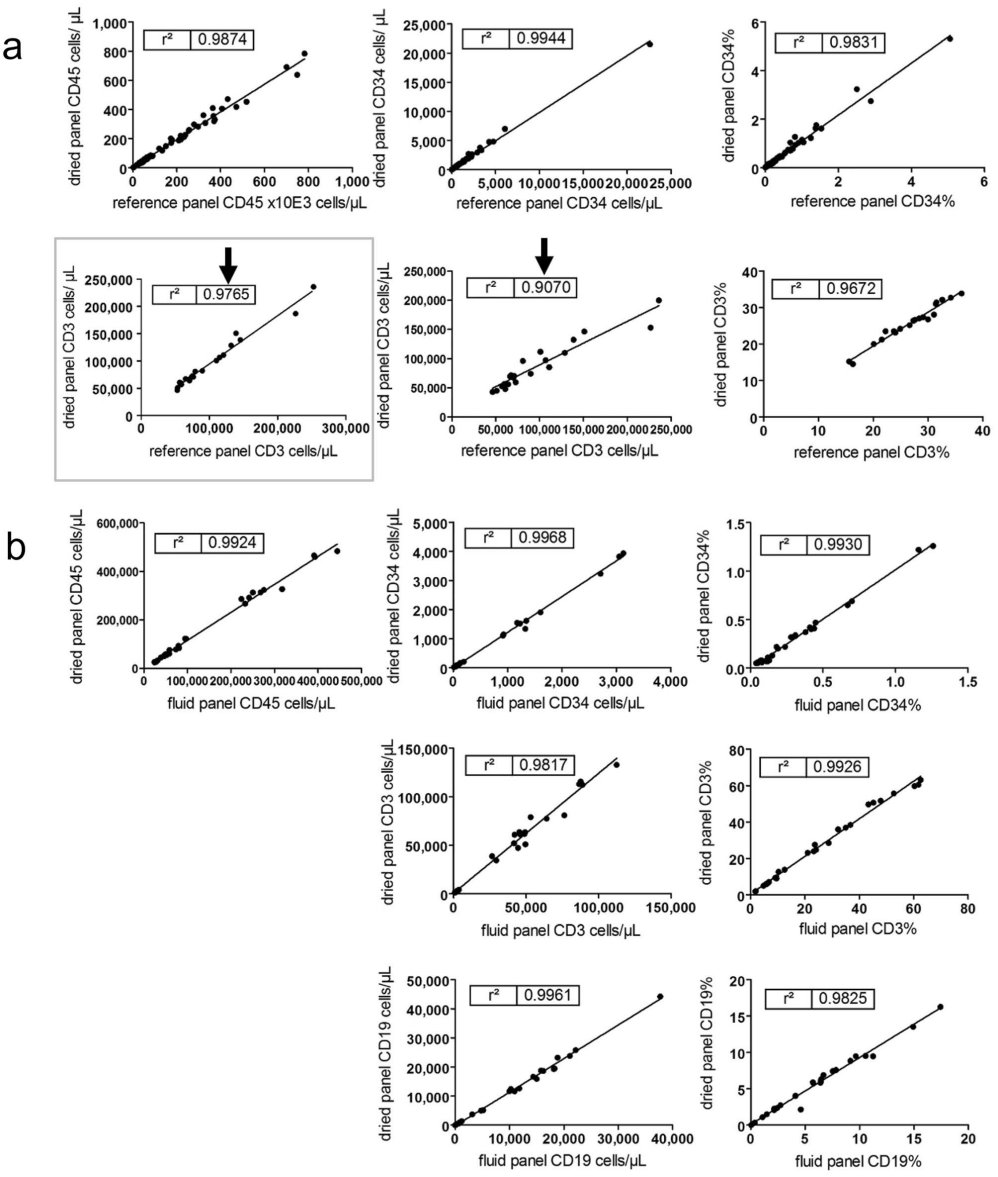
Validation results for accuracy testing. (**a**) Comparison the counting results of stem cells and T cells between the pre-formulated dried reagent panel including CD45 FITC, CD34 PE, 7-AAD, CD3 PB and CD19 APC and counting beads as well as the reference method using separated CD34 and CD3 determinations and the fluidic antibodies CD45 FITC, CD34 PE and CD45 FITC, CD3 PE each with 7-AAD and true count beads (autologous specimens only CD34 enumeration). Standard allogenic and autologous stem cell apheresis (n = 33) and mobilized whole blood (n = 35) of donors and patient after mobilization were compared by linear regression regarding the results of absolute counts in cells/µL (CD45 × 10^3^, CD34, CD3) and frequencies in % (CD34, CD3). (**b**) Accuracy of the measurement of the pre-formulated dried reagent panel and similar fluidic reagent panel (CD45 FITC, CD34 PE, 7-AAD, CD3 PB, CD19 APC and counting beads) were compared as described under (**a**) (LA of all cohorts: n = 17), whole blood of mobilized donors and patient (n = 11) including stem cells, T and B cells.

To exclude a systematic bias, we performed accuracy tests in concordance with the national External Quality Assessment (EQA). Results obtained using the pre-formulated tube were compared with the results obtained with validated methods on the same sample material. In brief, the results of the pre-formulated dried reagent panel tube were compared with the results of accepted methods using liquid antibody conjugates to enumerate stem cells (CD45 FITC, CD34 PE, 7-AAD, counting beads) and T cells (CD45 FITC, CD3 PE, 7-AAD, counting beads) separately (Fig. 2a). The statistical evaluations showed a strong correlation with r^2^ = 0.9672–0.9944 for the absolute cell counts of CD45 as well as for the frequencies and absolute counts of CD34 cells. Moreover, the r^2^ were higher when analyzing CD34 and CD3 cells in one tube (Fig. 2b) than in separate tubes (Fig. 2a). However, the regression coefficient between the dry pre-formulated reagent panel and the separate liquid T cell enumeration was lower for CD3 frequency (r^2^ = 0.9672) than for the CD34 frequency (r^2^ = 0.9831) and the acceptance criterion of r^2^ ≥ 0.95 for CD3 cells/µL (r^2^ = 0.9070) was not met. Further evaluations showed that when applying the relative percentage of CD3 to the CD45 absolute cell count from the CD34 determination demonstrating a measurement in one tube like the new assay, the r^2^ improved from 0.9070 to 0.9765 (Fig. 2a). The acceptance criteria fulfilled when re-calculating the CD3 absolute cell count. Further, the comparison with the similar panel based on liquid reagents (CD45 FITC, CD34 PE, 7-AAD, CD3 PB, CD19 APC, counting beads; Fig. 2b) showed a high degree of correlation between r^2^ = 0.9817–0.9968 for all frequencies and absolute cell counts. Likewise, the method with the new pre-formulated dried reagent panel and the same fluid reagent panel as stem kit with the drop-in of CD3 PB and CD19 APC successfully met the national EQA (INSTAND e.V., Germany) for stem cells as well as for T and B cells during the investigation period of two years.

We demonstrated the accuracy of the new approach. The comparison of the pre-formulated dried reagent panel with the separate CD34 and CD3 determination reference methods with liquid reagents, showed the advantages of determining stem cells and T cells in one tube. This finding has been confirmed by using the similar assay as liquid variant. The validation data with the pre-formulated dried reagent panel were submitted and approved as release testing assay by the Paul Ehrlich Institute (PEI) authority. This approval enabled us to use the new assay for LA products as well as for manufacturing of ATMPs.

### Optimized gate template results in low inter-operator variability

The inter-operator analysis provides an indication of the variability of the assay caused by the operator. The results of the Inter-Operator analysis are summarized in Table 4.

**Table 4:** Results of inter-operator analysis: operators (B to E) were checked versus the reference values of five several specimens analyzed with the pre-formulated dried reagent panel by Operator A.

Specimen	Parameter	Operator A (reference)	Operator B	Operator C	Operator D	Operator E	Average	Standard deviation	CV %
WB (healthy donor)	CD45 cells/µL	4497	4438	4446	4426	4431	4448	28.6234	0.64
	CD34 cells/µL	4	4	4	4	4	4.00	0.0000	0.00
	CD34%	0.08	0.08	0.08	0.08	0.08	0.08	0.0000	0.00
	CD3 cells/µL	1818	1801	1804	1796	1798	1803	8.7063	0.48
	CD3%	40.58	40.58	40.58	40.58	40.58	40.58	0.0000	0.00
	CD19 cells/µL	256	253	254	252	253	254	1.5166	0.60
	CD19%	5.70	5.70	5.70	5.70	5.70	5.70	0.0000	0.00
WB (mobilized donor)	CD45 cells/µL	33,770	36,464	36,070	36,054	36,022	35,676	1080.6961	3.03
	CD34 cells/µL	46	45	46	44	44	45	1.0000	2.22
	CD34%	0.13	0.12	0.13	0.12	0.12	0.12	0.0055	4.42
	CD3 cells/µL	2129	2120	2078	2077	2077	2096	26.0327	1.24
	CD3%	5.79	5.82	5.76	5.76	5.77	5.78	0.0255	0.44
	CD19 cells/µL	1162	1153	1139	1138	1137	1146	11.1669	0.97
	CD19%	3.16	3.16	3.16	3.16	3.16	3.16	0.0000	0.00
LA (non- mobilized donor)	CD45 cells/µL	78,650	78,392	76,708	76,440	76,310	77,300	1127.5114	1.46
	CD34 cells/µL	42	44	46	40	42	43	2.2804	5.33
	CD34%	0.05	0.06	0.06	0.05	0.06	0.06	0.0055	9.78
	CD3 cells/µL	45,984	45,932	44,960	44,802	44,726	45,281	624.2029	1.38
	CD3%	58.46	58.59	58.61	58.61	58.61	58.58	0.0654	0.11
	CD19 cells/µL	7208	7154	6970	6944	6952	7046	125.4225	1.78
	CD19%	9.16	9.13	9.09	9.09	9.11	9.12	0.0297	0.33
LA (mobilized donor)	CD45 cells/µL	297,140	291,520	280,860	281,280	284,670	287,094	7054.3022	2.46
	CD34 cells/µL	4760	4540	4390	4400	4450	4508	152.8725	3.39
	CD34%	1.60	1.56	1.57	1.57	1.57	1.57	0.0152	0.96
	CD3 cells/µL	69,710	68,860	66,140	66,230	67,020	67,592	1611.2324	2.38
	CD3%	23.47	23.64	23.55	23.55	23.55	23.55	0.0602	0.26
	CD19 cells/µL	24,440	23,940	23,040	23,070	23,350	23,568	606.8525	2.57
	CD19%	8.23	8.21	8.20	8.20	8.20	8.21	0.0130	0.16
LA (mobilized patient)	CD45 cells/µL	187,600	186,970	188,260	185,760	185,200	186,758	1,268.1956	0.68
	CD34 cells/µL	1330	1290	1340	1280	1280	1304	28.8097	2.21
	CD34%	0.71	0.69	0.71	0.70	0.69	0.70	0.0100	1.43
	CD3 cells/µL	11,990	12,080	11,660	11,460	11,470	11,732	289.6032	2.47
	CD3%	6.39	6.46	6.19	6.19	6.19	6.28	0.1311	2.09
	CD19 cells/µL	4400	4180	4080	3980	3940	4116	184.0652	4.47
	CD19%	2.35	2.24	2.17	2.15	2.13	2.21	0.0896	4.06

The CVs% between Operators were lower than 5% for all parameters except the parameter CD34% in the non-mobilized LA. This demonstrated a high consistency of the assay due to the optimized gate template.

### The pre-formulated panel allows the characterization of advanced cellular products

**Figure 3:**
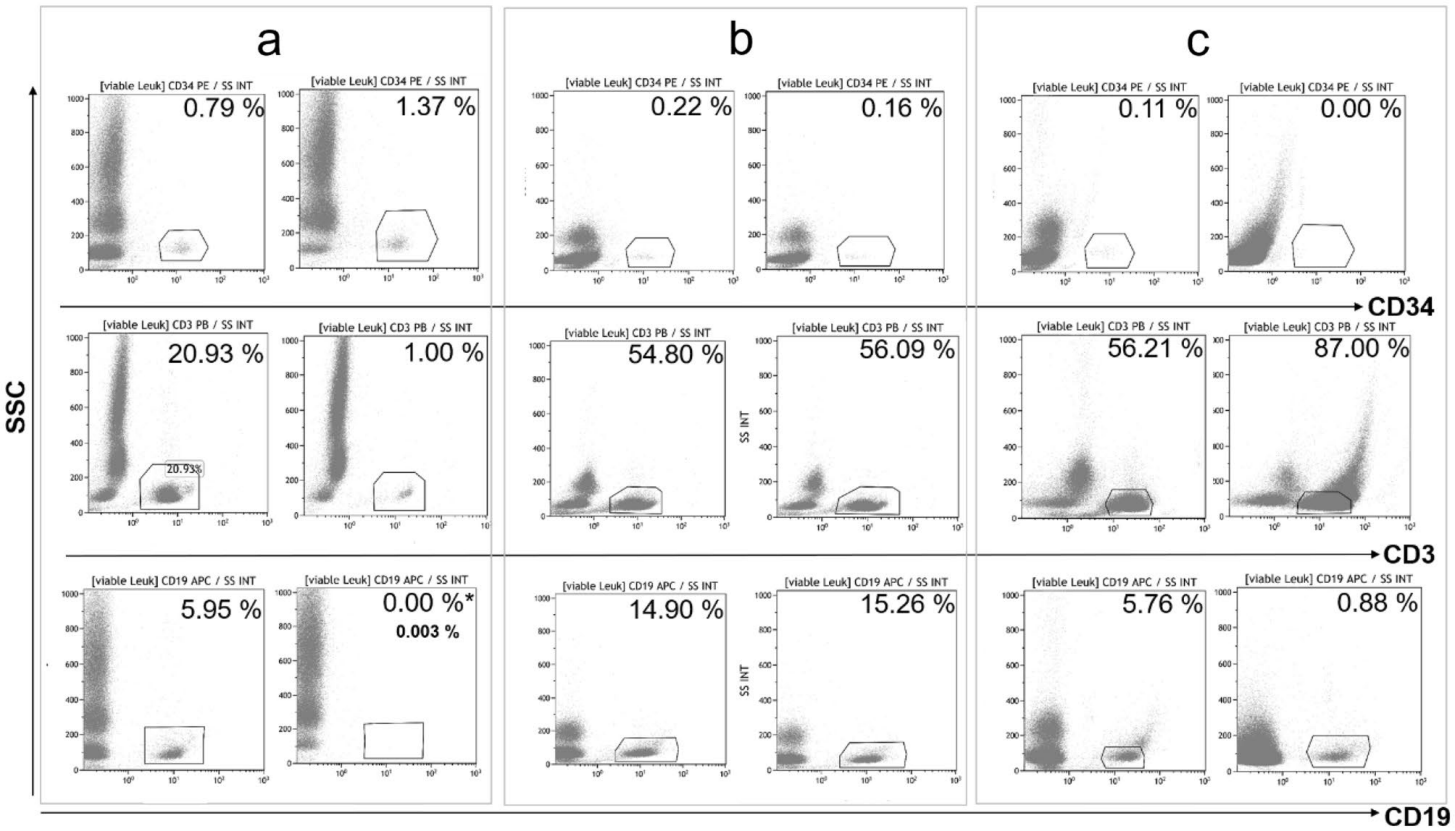
Application examples (each n = 1 before and after the manufacturing step) for the pre-formulated dried reagent panel with CD45 FITC, CD34 PE, 7-AAD, CD3 PB, CD19 APC and counting beads after method validation of standard stem cell products and donor lymphocytes. The samples were analyzed each as double approach. (**a**) CD19-TCR α/β-depletion: A mobilized stem cell apheresis of a heathy donor was incubated with magnetically antibodies against CD19 and TCR α/β-antigens from Miltenyi Biotec and depleted with the CliniMACS System. The assay was tested with cells before (part **a**, left) and after CD19-TCR α/β depletion (part a, right). CD19- and TCR α/β-depleted cells showed an enrichment of CD34 cells and the depletion of T-cells with TCR α/β character. Because of masked CD19 surface antigen (*) target cells were additional analyzed with the recommended fluorescence antibodies by Miltenyi Biotec (target: 0.003%). (**b**) Mononuclear cells for extracorporeal photopheresis (ECP): Apheresis cells of a non-mobilized healthy donor (part **b**, left) were diluted with isotonic NaCl-solution and mixed with Methoxsalen before UVA irradiation (part b, right). (**c**) Starting materials for CAR T cell manufacturing: The example shows the plots of the apheresis cells of a heathy non-mobilized donor before (part **c**, left) and after immunomagnetic CD4/8 labeling and selection (part **c**, right) with the prodigy system from Milenyi Biotec.

New selection processes and the production of novel ATMPs require a combined determination of stem cells, T and B cells under standardized conditions. This comprehensive analysis is also necessary (i) for characterizing the starting materials for ATMPs, (ii) for process control, and (iii) for cell expansion monitoring. While the above reported validation study of our pre-formulated dried reagent panel demonstrated its valid application in standard products like autologous and allogenic stem cell apheresis and donor lymphocytes, we also wanted to investigate the possibility of utilizing the same panel to analyze other products and ATMPs. Therefore, the pre-formulated panel was tested for three different cellular products: allogenic CD19-TCR-α/β-depleted stem cells, mononuclear cells for ECP therapy and CAR T cells in process validation from healthy donors (Fig. 3).

Allogeneic CD19-TCR-α/β-depleted stem cells were measured before and after CD19- and TCR α/β-depletion with the CliniMACS System (Miltenyi Biotec, Bergisch Gladbach). After depletion an enrichment of CD34 cells from 0.79 to 1.37% of total viable cells was achieved while a reduction of CD3 cells from 20.93 to 1.00% of total viable cells was observed (Fig. 3a). After labeling with immunomagnetic beads, the common issue with the masking of CD19 antigens by the manufacturing antibody occurred. In fact, the pre-formulated dried reagent panel with anti-CD19 APC could not detect the B cell population anymore.

To determine the actual CD19 value, all fractions were analyzed with the recommended fluorescence antibodies by Miltenyi Biotec, Bergisch Gladbach and we observed an CD19 depletion from 5.95% to 0.003%. Figure 3 shows the analysis of cells before and after manipulation with methoxsalen without UVA irradiation in the context of ECP product manufacturing with the pre-formulated dried antibody panel (Fig. 3b). We could demonstrate that the treatment with methoxsalen did not influence T and B cell frequencies.

In a third experiment, starting materials for CAR T cell manufacturing were analyzed before and after CD4/CD8-selection using immunomagnetic beads (Fig. 3c). The obtained target fraction after selection did not contain any CD34 cells and the T cells were enriched. The frequency of CD19 cells was reduced from 5.76% to 0.88% and the CAR T cell culture via Prodigy system (Miltenyi Biotec, Bergisch Gladbach) was started. Our results show that the pre-formulated reagent panel can be used for in-process controls during the cell separations steps or ATMP manufacturing. Nevertheless, additionally validation steps are necessary for analyzing the influence on cells of immunomagnetic beads and matrices before applying for an officially approved release test.

### Additional markers can be easily added to the dried panel assay

**Figure 4:**
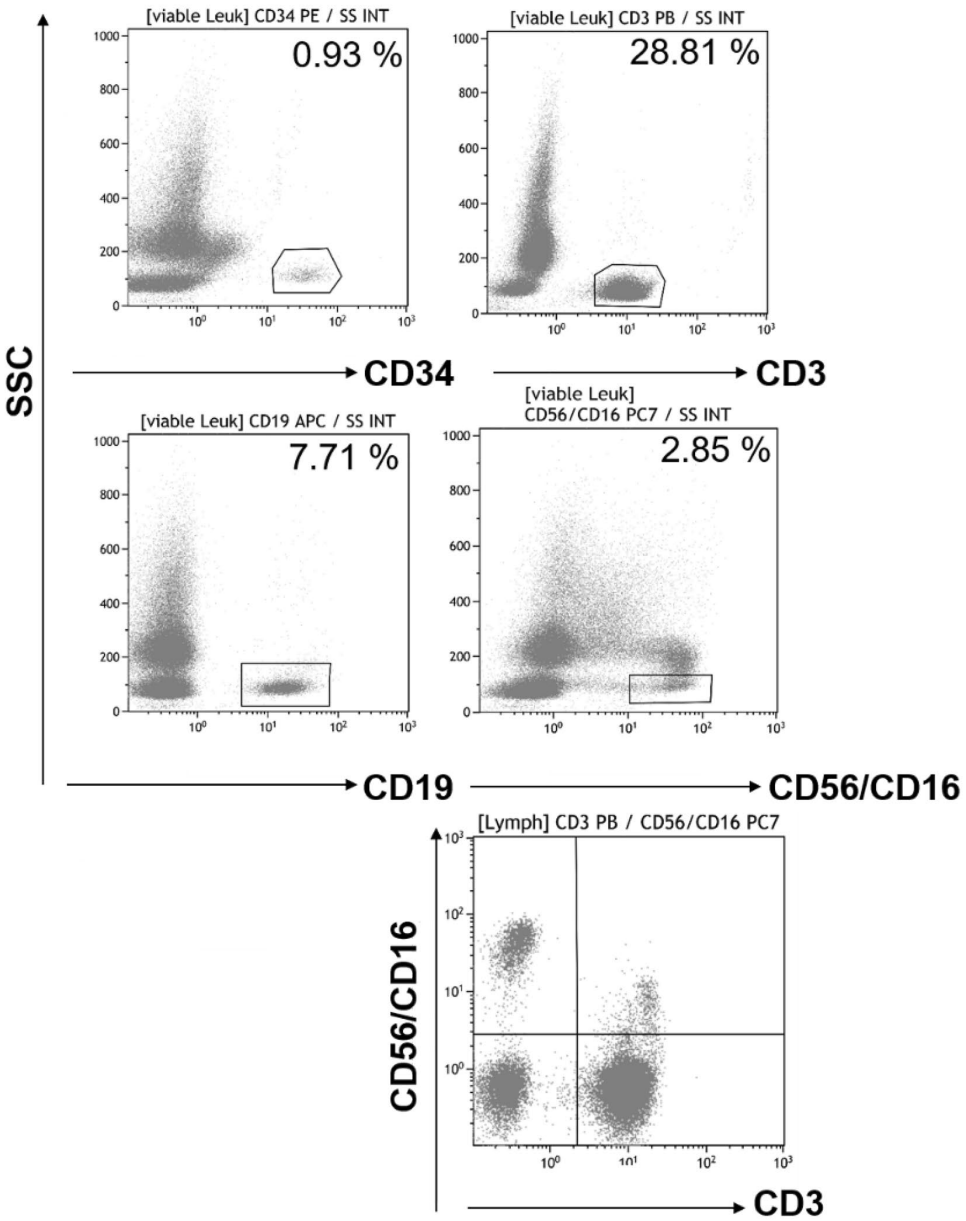
Expansion of the pre-formulated dried reagent panel: The template protocol (Supplemental Fig. S4) can be expanded for further parameter testing. For example, the addition of CD16 PC7 and CD56 PC7 fluorescence conjugates as drop-in to the dried pre-formulated tube enables the quality control for manufacturing of products enriched with natural killer cells (NK: CD45^+^/CD56^+^/CD16^+^/CD3^−^) including natural killer T cells (NKT: CD45^+^/CD56^+^/CD16^+^/CD3^+^) gated versus side scatter by excluding CD16^+^ neutrophils and CD16^+^ monocytes in the viable CD45^+^ leucocytes gate (Supplemental Fig. S4) for absolute counting. To distinguish between NK and NKT plotting CD3 PB versus CD16/56 PC7 gated on the lymphocyte population is crucial**.**

The multi-color flow cytometer Navios with three lasers enables the detection of up to 10 fluorescent parameters. Thus, the detection of more leucocytes subpopulations is possible on this platform by adding respective antibodies to the pre-formulated reagent panel. For example, it is possible to determine the populations of NK cells and natural killer T (NKT) cells by detecting the surface antigens CD56 and CD16 in combination with the T cell marker as shown in Fig. 4.

The addition of the liquid antibodies for NK cell determination changed the analysis method with the pre-formulated reagent panel and new validation measures were required. The supplementary validation with reference material showed reproducible and valid results. All measurements (n = 30) were within the reference range of the reference blood regarding the absolute values of WBC (6000–7600 cells/µL), CD34 (0.7–5.3 cells/µL), CD3 (1542–2242 cells/µL) and CD19 (134–434 cells/µL) as well as CD3/CD16/CD56 (144–434 cells/µL). The acceptance criteria for linearity were fulfilled between the concentrations 527–7056 CD45 cells/µL (r^2^ = 1.0000), 129–1842 CD3 cells/µL (r^2^ = 0.9998), 23–297 CD19 cells/µL (r^2^ = 0.9998), 16–226 CD16^+^/CD56^+^/CD3^−^ cells/µL (r^2^ = 0.9999) and 7–88 CD16^+^/CD56^+^/CD3^+^ cells/µL (r^2^ = 0.9867) (Supplemental Fig. S5). The low CD34 concentrations in the reference material did not allow the assessment of detection linearity. The repeatability assay showed a small variation coefficient between 0.80 and 2.16%, except for CD34 cells (invalid results regarding the Limit of Quantification (CD34 cells/µL: 2, CD34%: 0.04; Supplemental Table S6). The LOQ for NK cells was 2 cell/µL (Supplemental Table S7).

The expansion of the pre-formulated dried reagent panel with the help of the drop-in antibody conjugates to detect further populations of interest like NK cells and NKT cells is possible, but must be validated accordingly to the respective cellular products when used as drug release test.

## Discussion

The validation of release testing methods for pharmaceutical manufacturing is subjected to very strict regulatory requirements. Here, we present for the first time a novel pre-formulated panel based on dried antibodies designed for the comprehensive characterization of cellular products with the idea to reduce sources of human error and variability. The validation of this optimized stem cell enumeration panel follows the ISHAGE analysis protocol and fulfills the current regulatory standards as it has been approved by the PEI^1,13^. Moreover, due to the broad applicability of the newly developed assay, its validation was preceded by an extensive risk assessment and was developed in agreement with the ICH Quality Guidelines^13^.

In fact, our extended product release assay is not only based on the historical ISHAGE protocol, but it also combines the enumeration of viable stem cells with T and B cell counts. The fluorescently labeled antibodies for the immunophenotype-based detection as well as the viability dye and the counting beads used in this assay are no longer in liquid format like in the CE (Conformité Européenne) certified stem cell enumeration kits available on the market^14^, but are provided in a unitized dried format as a layer in the bottom of the assay tube. Based on this modern one tube design, this assay is highly reliable and broadly applicable, and it reduces the risk of operator failure. Furthermore, it can be used for a great variety of cellular products such as autologous stem cells, enriched cellular products, lymphocyte-containing fractions, bone marrow and ATMPs.

The results of the validation of our pre-formulated tubes are in line with the previously determined reference values from validation studies based on the ISHAGE protocol. The laboratory range for the CD34 absolute counts in fresh G-CSF mobilized WB was higher with the pre-formulated reagent panel than the range determined by Dauber et al. with standard liquid kits for stem cell counting^14^. The determined range for CD34 frequency among CD45 cells in all sample types common in stem cell characterization (e.g. mobilized peripheral blood, fresh and frozen LA, cord blood and bone marrow) showed a lower maximum value (7.87%)^14^ than the maximum value determined with our dried panel (11.48%). This observation underlines the need for stem cell laboratories to handle their own sample material with specific characteristic. Every laboratory should therefore determine its own internal laboratory range. In our study, sample materials from allogenic donors and also from patients undergoing autologous stem cell therapy were included in the internal laboratory range. We determined the same LOQ as Massin et al.^15^ in the validation study with the BD stem cell enumeration kit. Our linear measuring range for high and low CD34 absolute counts determined with the pre-formulated dry reagent panel, is 20 times higher than the linearity range found by Massin et al*.*^15^ and therefor extends the reference values for standard stem cell analysis according to the ISHAGE protocol.

Some manufacturing facilities use automatic hematology analyzers to determine the stem cell count prior to stem cell apheresis. The sensitivity of the pre-formulated tube is higher than the sensitivity of the hematology analyzer (CD34 cells/µL: 8.9)^16^. Interestingly, compromised stainability of CD34 cells with the hematology analyzer were reported among myeloma patients due to the unspecific labelling^16^. The validated assay with the pre-formulated tube detects unspecific bindings via the isoclone control allowing to correct the stem cell count accordingly by subtracting the unspecific binding from the CD34 value. This explains the higher accuracy of our assay.

Our reference values for CD45, CD3, CD19 cells in the WB of healthy donors were in agreement with the results of the ONE study with the validated pre-formulated dried tube and their absolute count^17,18^. A direct comparison of cell frequencies values was not possible due to the different gating strategies. In detail, our gating strategy relates frequencies to all leukocytes in apheresis cell products and not to lymphocytes in WB as the gating strategy of the study mentioned above. Besides this, all dead cells can be excluded by viability staining to control transport and storage^19^, resulting in an improved accuracy of the measurement.

Our mid- to long-term goal, was to go beyond the optimization of the ISHAGE protocol^1–5^, in order to use this gating strategy for absolute CD4 T lymphocytes counting^20^ and absolute viable T cells assessment in cellular products^21^. Finally we wanted to combine the quantification of both, stem cells and further cell populations. Strobel *et al.* combined the ISHAGE protocol with a CD3 determination using a liquid reagent panel, whereby the bead-based quantification of the CD3 determination performed better than the volumetric quantification on the flow cytometer^22^. In addition to the stem cell and T cell population, the pre-formulated dried reagent tube used in our approach allows to determine the B cells with the help of bead counts.

The cell populations frequencies measured with the pre-formulated dried reagent panel are crucial for the manufacturing and therapeutic application of different cellular products. For example, the assessment of CD34 cells across different ranges is of high value for the clinical routine. In fact, patients and donors cannot undergo stem cell apheresis until the CD34 cell counts exceed a minimum levels (peripherally ≥ 5 to 15 CD34 events/µL)^23,24^. Moreover, high numbers of CD34 counts are present in enriched apheresis products and their accurate assessment is crucial for the stem cell transplantation outcome. For further characterization, the dose of multipotent progenitors among mobilized high stem cell can be phenotypically determined since multipotent progenitors might impact engraftment and immune reconstitution after stem cell transplantation^25^. Moreover, the use of an 8-color pre-formulated dried panel for the detection of minimal residual disease (MRD) was validated and its use can increase the quality of autologous blood stem cell products from myeloma patient apheresis to avoid the infusion of a residual disease^26^.

The composition of LA used as starting material for genetically engineered ATMPs is critical. Many of the manufactures perform a T cell selection in order to reduce the risk of administering manipulated stem cells into the patient^27,28^. Our validation showed that unstimulated LA from healthy donors had higher CD34 cells levels (n = 20; average: 0.09%; min: 0.01%; max: 0,24%; median: 0.07%) compared to the reference value of 0.05% in WB and a lower CD3 absolute cell count. The quantification of viable lymphocyte subpopulations for T cells, NK cells, B cells and monocytes in unstimulated LA (mononuclear cells) was validated by Mfarrej et al*.* with three standard tubes each with separate CD45 staining and counting beads as liquid antibody panels^29^. Our pre-formulated antibody panel tube was developed to support a broad application for cellular products including stem cell quantification. Our validated assay allows cell counting of the CD34, CD3, CD19 cell population in a single tube with all populations related to the CD45 population and quantified using. This ensures a more consistent product characterization.

In haploidentical stem cell transplantation, T cell depletion is crucial for both the success of the treatment and the clinical outcome. Acceptance limits for T cell contamination must be specified, due to the associated threat of GvHD^8,30^. The T cell analysis following depletion, may be difficult to perform due to the low levels of T cells and because of the increased autofluorescence and unspecific binding of manipulated cells^8,30^. Over the time, the approach to haploidentical stem cell transplantation has changed and the initial CD34 cells selection has been replaced by a CD19 and TCR α/β-depletion^30–32^. The higher CD3 absolute count is now easier to detect because the remaining T cell population (CD3^+^/TCR y/d^+^) is around 1% after depletion (Fig. 3, CD19 TCR α/β-depletion). Similar to stem cells, the pre-formulated reagent panel can be used to reliably determine high and low T cell levels.

Finally, our enumeration panel can be also used during the manufacturing process of CAR T cells. In fact, the determination of the T cell dose expressing the specific CAR is conducted several times on several manufacturing days for in-process control and also for the final testing^33,34^. The absolute T cell count, as determined with the pre-formulated dried panel, together with the assessment of the frequency level of CAR-expressing T cell cells with a dedicated assay, can be combined to calculate the actual CAR T cell dose contained in a cellular product. The drop-in concept would also enable the analysis of starting material for NK cell based ATMPs^9,35^. Furthermore, the increased application of anti-CD19 CAR therapies warrant adequate release testing that includes CD19-B cells^36^.

In summary, we have developed a pre-formulated panel tube to address the growing regulatory requirements of release testing. The design of this new analytical test enables a significant reduction of pipetting time thereby enabling a detailed and reliable analysis. We also have put a focus on regulatory challenges like the production of batch sizes of up to 2000 tests of the pre-formulated dried tube. The tubes can be simlpy stored at room temperature and have a long shelf-life of two years after manufacturing. The release testing with the pre-formulated tubes represents an advancement over the fluidic CE certified kits because besides the stem cells, T cells an B cells can be analyzed in parallel in compliance to the ISHAGE protocol. The dried pre-formulated tubes are widely applicable across a variety of standard cellular products with or without stem cells and starting cells for ATMPs and ATMPs. Furthermore, our method is more time efficient and reduces operator failure. In summary, a respective improved standardization can further support an increased product quality and ultimately improved cell therapy approaches for patients.

## Methods

### Instrument acceptance testing and risk assessment under good manufacturing practice (GMP)

Release testing of the Navios flow cytometers (Beckman Coulter, France) was preceded by risk analysis using the Failure Mode and Effects Analysis. Based on written qualification plans the flow cytometers underwent validation and QC procedures complying with standard specification and defined requirements. All processes in the QC Laboratory follow the principles of European Pharmacopoeia, GMP guidelines and ISO15189 standards. All activities were described in standard operation procedures and the laboratory staff was adequately trained by application specialists and internal training programs. Validation plans used for the procedures described here followed relevant validation guidelines for analytical methods^13,37,38^.

### Summary of the validation study and specimen collection

The validation measurements in analytics, for example, linearity, sensitivity as well as accuracy were performed according to the ICH Q2 (R1) guidelines^39^. The study was approved by the Ethics Committee of the Charité—*Universitätsmedizin* Berlin, Germany (EA2/129/19). We confirm that all methods were performed in accordance with the relevant guidelines and regulations. The informed consent was obtained from all subjects and/or their legal guardian(s). Specimens of healthy donors (cohort 1: not-mobilized, cohort 2: G-CSF mobilized) and patients suffering from multiple myeloma and a wide range of lymphomas (cohort 3: G-CSF-mobilized) were investigated including WB, mobilized WB before and after LA as well as cellular products. WB was collected into vacutainers (Becton Dickinson, Germany) containing the anticoagulant EDTA. LA samples were collected with the Spectra Optia Apheresis System (Terumo BCT Europe N.V.) Furthermore, cryopreserved autologous QC apheresis samples were analyzed. Unless otherwise specified the viable cell parameters (%, cells/µL) were statistically evaluated with GraphPad Prism 5 (GraphPad Software V5.01, USA).

### Sample preparation and staining procedures

White blood cells (WBC) were determined by the automated hematology analyzer XP-300 (Sysmex, Germany) and if necessary diluted with phosphate buffered saline without calcium- and magnesium chloride (PBS, Gibco, Germany) and 0.5% bovine serum albumin (BSA, Sigma Aldrich, Germany). In case of cryopreserved samples, a minimum of 1 to 10 dilutions in PBS/10% human serum albumin (HSA, Shire, Germany) was applied. A defined volume of WBC per sample (≤ 3 × 10^6^ cells, 100 µL) was applied using the reverse pipetting approach.

The tubes contained dried reagents consisting of fluorescence antibodies conjugates (Isotyp IgG1) CD45 FITC (Clone J33), CD34 PE (Clone 581.1.5), CD3 PB (Clone UCHT1) and CD19 APC (Clone J3.119), the dead marker dye 7-aminoactinomycin D (7-AAD) as well as counting beads. The tubes were manufactured by Beckman Coulter (France) using the DURAClone dry technology. After 15 min incubation of the validation samples at room temperature in the dark, red blood cells were lysed with 2 mL 1 × IO-lyses buffer (Beckman Coulter) for 10 min. The same pre-formulated reagent panel including also the unlabeled CD34 antibody (Clone 581.1.5) was used as internal isoclone control. In order to establish the count of NK cells the liquid antibodies CD16 PC5 (Clone 3G8) and CD56 PC5 (Clone N901 (NKH-1) were added (each 10 µL) to the pre-formulated dried reagent panel before pipetting the sample. Reference methods were used to compare the new assay: The similar antibody cocktail as liquid variation includes the Stem Cell Enumeration Kit (CE/IVD-Kit, Beckman Coulter) with the antibodies CD45 FITC (Clone J33)/CD34 PE (Clone 581.1.5), CD45 FITC/isoclonic control-PE and 7-AAD for cell staining, the lyses solution as well as stem count fluorosphores. The staining procedure followed the instructions of the kit and, in addition, the antibodies CD3 PB (Clone UCHT1; Beckman Coulter) and CD19 APC (Clone J3.119; Beckman Coulter) were used for staining the T- and B-cells (each 10 µL). After 20 min the 1 × NH_4_ red blood lyses solution was added for further 10 min. Finally, a known number of stem count fluorosphores was added.

Further reference measurements were performed as separate CD34 and CD3 determinations with the FACS Calibur (Becton Dickinson, Germany). For staining, 20 µL of each liquid fluorescence antibodies (Isotyp IgG1) CD45 FITC (2D1, CE, Becton Dickinson, Germany), CD34 PE (Clone 8G12, Becton Dickinson) for CD34 determination and CD45 FITC, CD3 PE (SK7, CE, Becton Dickinson) for CD3 determination was added to true count beads tubes (Becton Dickinson). In all measurements the viability was checked by staining with 7-AAD (BD Pharmingen, Europe).

### Sample acquisition

The Navios underwent daily verification of optical alignment and fluidics using further fluorescent microspheres (Flow Check beads, Beckman Coulter) and reference blood (stem cell control kit, Becton Dickinson; CDChex Plus, Streck Corporate, Nebraska). The flow cytometer settings were created with the auto-setup function of the Navios software using single stained target cells in the corresponding fluorescence channels and the setting was verified by multi stained target cells. As stop criteria the number of 75,000 CD45^+^ events and in case of stem cell analysis 100 CD34^+^ events per sample were determined. One thousand counting beads were needed to determinate the absolute cell count.

### Data analysis

Data analysis was performed using the Navios software with a pre-defined analysis template that requires only fine adjustment by the operator. Cells were analyzed by using the fluorescence antibody conjugates and the death versus side scatter plot (SS INT). Supplemental Figure S4 explains the gating strategies for the enumeration of stem cell, T and B cells. The absolute count was calculated by the known numbers of counting beads in correlation to the count of stem cell, T and B cell populations. Viability was estimated by the division of the viable cells by the total number of cells (dead and viable cells).

### Laboratory range

The new measurement method was applied to common samples from the manufacturing process, whereby a measurement range for the QC laboratory of the relevant parameters was determined. This included cell therapy products and starting material for further processing like cryopreservation and selection processes (non-mobilized donors: n = 20, mobilized donors: n = 30, mobilized patient: n = 30 + 30) as well as WB of healthy non-mobilized donors and WB before and after the LA process in autologous or allogenic settings (non-mobilized donors: n = 15, mobilized donors: n = 30, mobilized patient: n = 30). Results were assessed by the statistical parameter min, max and median. No acceptance criteria were defined.

### Linearity

Linearity was tested in the specimen WB (mobilized donors and mobilized patient) as well as apheresis cells of all cohorts after serial dilution with a minimum of 7 concentrations (dilution factor 1 to 128). The estimated cell concentration was compared with the expected values as regression analysis. The acceptance criteria were r^2^ ≥ 0.95.

### Sensitivity

Two approaches to sensitivity determinations were used to calculate values for Limit of Detection (LOD) and Limit of Quantification (LOQ). The established approach determined the LOD and LOQ with the help of 10 replicates of a low-concentration sample of the parameter to be observed. The derivative of the standard deviation multiplied by a factor of three determined the LOD whereas the LOQ was determined by multiplying the standard deviation by factor 10. The novel approach according to Armbruster et al. enables the further determination of the Limit of Blank (LOB)^40^, whereas the standard deviation (SD) of the low concentration sample was already been determined by the established approach and used for statistical evaluation of the LOD. No acceptance criteria were predetermined. Reference samples with low parameter concentration were used for the LOD determination whereas the determination of LOB needs reference samples with lack of the parameter. The reference sample with the low CD34 level and without CD34 cells were measured in the reference laboratory as part of the CD34 monitoring finding the optimal starting point for stem cell apheresis. The reference samples were obtained via blood collection from patients who has received chemotherapy and were on day 5 of G-CSF mobilization. Depending on the patient’s mobilization the CD34 concentration at this time is 0 to < 10 cells/µL. As low CD3 and CD19 level sample the target fraction of an CD34 selection was used. The human hematopoietic CD19 positive cell line Raji (ATCC, CCL-86) immunophenotypically lacks the CD3 surface antigen. Raji cells used therefore as reference cells to determine the LOB of the CD3 parameter. The cell line was cultivated in RPMI 1640 (Gibco Thermo Fischer Scientific, MA, USA) supplemented with 10% fetal bovine serum (Millipore Sigma, MA, USA) in a humidified atmosphere containing 5% CO2 at 37 °C. Identity was confirmed using short tandem repeat DNA genotyping (Eurofins Scientific SE, Luxemburg, Luxemburg). Cultures were checked regularly for mycoplasma contamination using the PlasmoTest Kit (Thermo Fischer Scientific, MA USA).

### Accuracy

The DURAClone based assay was compared with the established analysing methods for the determination of CD45, CD34 and CD3 cells. The comparison methods estimated the CD34 count and the CD3 count in different tubes without considering the CD19 parameter. Therefore, WB before and after LA (mobilized donors: n = 40; mobilized patients: n = 28) as well as fresh LA products and LA products after cryopreservation (mobilized donors: n = 20; mobilized patient: n = 45) were tested. Further accuracy tests were done with a fluidic reagent panel of the same composition (CD45 FITC, CD34 PE, 7-AAD, CD3 PB, CD19 APC) like the pre-formulated dried tube with similar specimens (all cohorts: n = 28). The absolute counts and frequencies were statistically analyzed by regression analysis with an acceptance criterion of r^2^ = 0.95. The new method with the pre-formulated dried reagent panel as well as the fluidic panel were tested with reference material from national EQA (Instand e.V., Germany) for the CD34 enumeration and CD3 and CD19 enumeration.

### Inter-operator-analysis

To determine the inter-operator variability of our assay, five samples (unstimulated/mobilized WB and LA) and five operators were examined. The samples measured and evaluated by operator A were considered as reference values. Operators B to G performed the re-gating on the measured data set (list mode analysis). The acceptance criteria were CV% ≤ 10% between the operator results.

### Panel expansion: short validation design

Reference blood (CD Chex Plus, Streck Corporate, Nebraska) was used to pre-test the expanded panel combination for NK cells and NKT cells. Because of blood stabilization cells were permeable for the death marker so the gating strategy was adjusted and 7-AAD + cells were included also. The material with the reference values enables anytime the proof of accuracy of undiluted as well as diluted replicate measurement. Linearity was tested after serial dilution into five concentrations (dilution factor 1 to 64) as triplicates. Estimated cell concentration was compared with the expected values by the acceptance criteria r^2^ ≥ 0.95. The repeatability was tested with five prepared tubes, whereas the first tube was measured five times. The sensitivity for CD16^+^/CD56^+^/CD3^−^ cells was measured by 1:128 dilution of the reference blood offered through the linearity approach.

## Supplementary Information

Supplementary Figure S4.

Supplementary Figure S5.

Supplementary Table S1.

Supplementary Table S2.

Supplementary Table S3.

Supplementary Table S6.

Supplementary Table S7.

## Data Availability

The datasets generated during and/or analyzed during the current study are available from the corresponding author on reasonable request.
