# Supplementary material for: Widely applicable, extended flow cytometric stem cell enumeration panel for quality control of advanced cellular products
Source: Sci Rep. 2022 Oct 26;12:17995. doi: 10.1038/s41598-022-22339-1 (PMC9605971; doi:10.1038/s41598-022-22339-1)
Supplement: Supplementary file 1 — Supplementary Figure S4. [file 41598_2022_22339_MOESM1_ESM.docx]

**Widely applicable, extended flow cytometric stem cell enumeration panel for quality control of advanced cellular products**

Katy Haussmann^1,*^, Mathias Streitz^2,3^, Anna Takvorian^1^, Jana Grund^1^, Zemra Skenderi^1^, Carola Tietze-Bürger^1^, Kamran Movassaghi^1^, Annette Künkele^1,4-7^, Agnieszka Blum^8^, Lars Bullinger^1,5,6,9^

^1^ Charité–Universitätsmedizin Berlin, corporate member of Freie Universität Berlin, Humboldt Universität zu Berlin, and Berlin Institute of Health, Stem Cell Facility, 10353 Berlin, Germany

^2^ Institute of Medical Immunology, Charité – Universitätsmedizin Berlin, corporate member of Freie Universität Berlin, Humboldt-Universität zu Berlin, and Berlin Institute of Health, Augustenburger Platz 1, Berlin, 13353 Germany

^3^ Department of Experimental Animal Facilities and Biorisk Management, Friedrich-Loeffler Institut, Greifswald-Insel Riems, Germany

^4^ Charité–Universitätsmedizin Berlin, corporate member of Freie Universität Berlin, Humboldt Universiät zu Berlin, and Berlin Institute of Health, Department of Pediatric Oncology and Hematology, 10353 Berlin, Germany

^5^ German Cancer Consortium (DKTK), 10117 Berlin, Germany

^6^ German Cancer Research Center (DKFZ), 69120 Heidelberg, Germany

^7^ Berlin Institute of Health at Charité - Universitätsmedizin Berlin, Charitéplatz 1, 10117 Berlin, Germany

^8^ Ardigen, 30-394 Kraków, Poland

^9^ Charité–Universitätsmedizin Berlin, corporate member of Freie Universität Berlin, Humboldt Universität zu Berlin, and Berlin Institute of Health, Department of Hematology, Oncology and Tumorimmunology, Charité – Universitätsmedizin Berlin, Berlin, Germany

**Supplemental Information**

The ISHAGE basic gating (Supplemental Figure S4, a) starts with the definition of single counting beads, total and viable cells as well as gating viable leukocytes regarding the SSC threshold which reduces the counting of cell debris and therefore the detection of unspecific binding. The plot “time versus beat count” (not shown) separates the singlet counting beads from total beads with duplicates so the optimal counting beads can be identified in the Plot FL1 versus FL2 (Supplemental Figure S4, a). With the help of the dead marker 7-AAD cells can be distinguish between total cells (the 7‑AAD^+^/^-^ cells) and viable cells (7-AAD^-^ cells; Supplemental Figure S4, a). Viable leucocytes were detected with the pan-CD45 marker in a polygonal gate to precisely discriminate residual red blood cells and cell debris (Plot SS INT versus CD45-FITC) and include the sub populations like stem cells and lymphocytes (Supplemental Figure S4, a). These basic gating steps allow the further determination of viable stem cells (CD45^+^/CD34^+^; Supplemental Figure S4, b) as well as T cells (CD45^+^/CD3^+^; Supplemental Figure S4, c) and B cells (CD45^+^/CD19^+^; Supplemental Figure S4, c) within the viable leucocytes amount (CD45^+^/7‑AAD^-^). Following the ISHAGE protocol and in order to prove the low expression, CD34 stem cells were back gated in the plot CD45 versus SS INT (Supplemental Figure S4, b). The CD45^+^_dim_/CD34^+^ cells and lymphocyte were then back gated to check the position in the forward scatter (FS) INT versus SS INT; Supplemental Figure S4, b) in comparison of the lymphocytes (Supplemental Figure S4, b) and exclude further cell debris.

In order to the stem cell populations the value of the CD34 isoclonic control was deducted from the CD34 value. The ISHAGE gating strategy results in the determination of the haematopoietic progenitor cells (HPC) used as value for stem cell therapy.


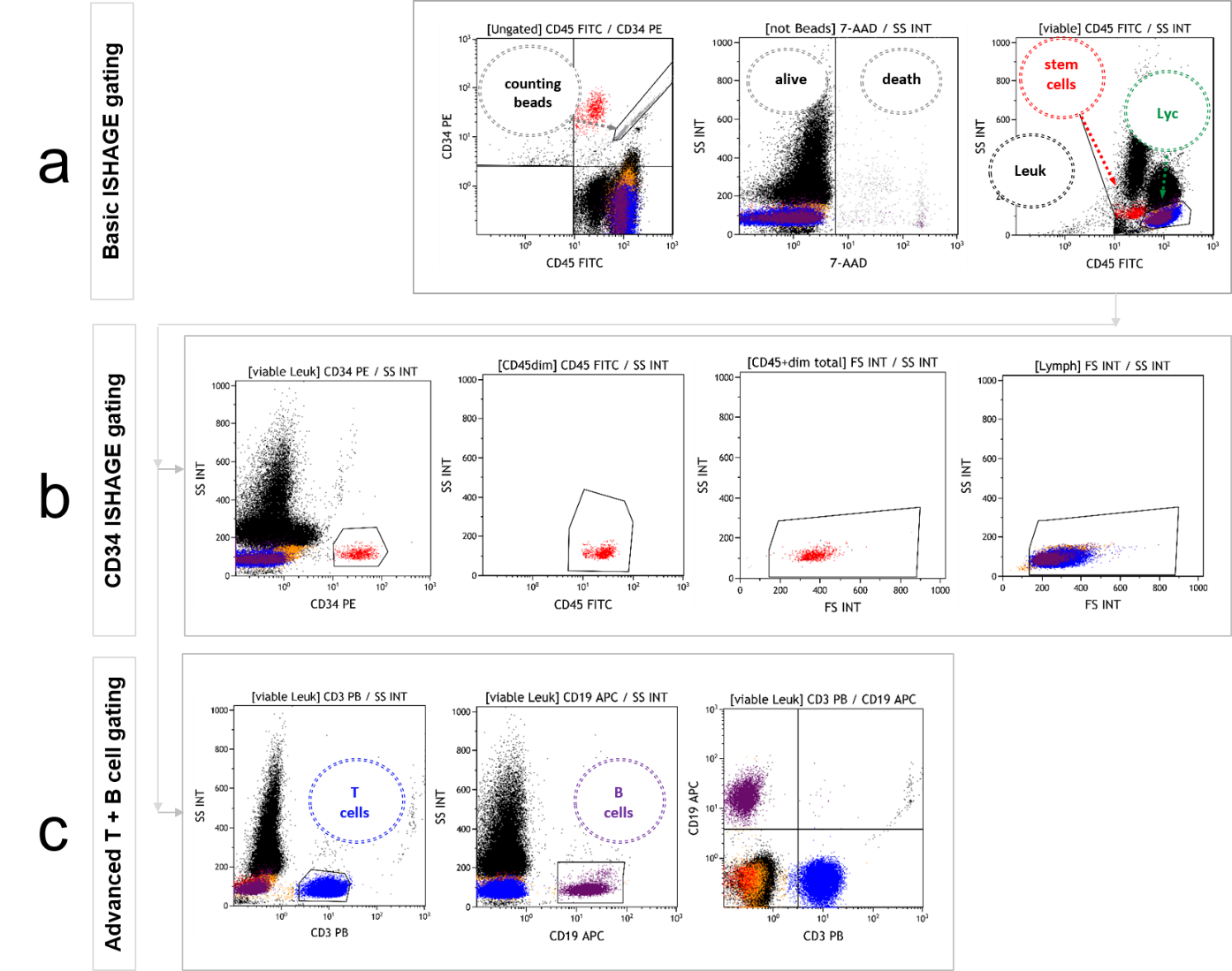


Supplemental Figure S4: Detailed analysis template based on ISHAGE protocol for enumeration of viable CD34/CD3/CD19/CD16/CD56 positive cells: After stem cell mobilization a healthy donor cell product was stained with the pre-formulated dried reagent panel including CD45 FITC, CD34 PE, CD3 PB, CD19 APC, 7-AAD and counting beads. a: Basic ISHAGE gating: After defining the gate for counting beads the basic gating starts with the differentiation of total cells (7-AAD^-/+^) and viable cells (7-AAD^-^) with the viability marker 7-AAD. The viable leukocytes (CD45^+^) were gated including the viable subpopulations of interests for example stem cells and lymphocytes including T and B cells. b: CD34 ISHAGE gating: Following the ISHAGE-protocol the stem cells (CD45^+^/CD34^+^) were analyzed against the Side Scatter (SS INT) and were backgated to prove the low CD45 expression of hematopoietic stem cells followed by excluding cell debris in comparison with the lymphocyte back gate in the forward and side scatter. c: Advanced T and B cell gating: T (CD45^+^/CD3^+^) and B cells (CD45^+^/CD19^+^) populations determined versus side scatter after checking general plausibility with the plot CD19 APC versus CD3 PB. Note: In general, all gates were optimized and fixed before in a template protocol for acquisition and only a fine adjustment was necessary to define counting beads and the populations of interest. In this fig. analysis was done with the Kaluza software (Beckman Coulter) for better representation of plots and cells.
