# Supplementary material for: Widely applicable, extended flow cytometric stem cell enumeration panel for quality control of advanced cellular products
Source: Sci Rep. 2022 Oct 26;12:17995. doi: 10.1038/s41598-022-22339-1 (PMC9605971; doi:10.1038/s41598-022-22339-1)
Supplement: Supplementary file 3 — Supplementary Table S1. [file 41598_2022_22339_MOESM3_ESM.docx]

**Widely applicable, extended flow cytometric stem cell enumeration panel for quality control of advanced cellular products**

Katy Haussmann^1,*^, Mathias Streitz^2,3^, Anna Takvorian^1^, Jana Grund^1^, Zemra Skenderi^1^, Carola Tietze-Bürger^1^, Kamran Movassaghi^1^, Annette Künkele^1,4-7^, Agnieszka Blum^8^, Lars Bullinger^1,5,6,9^

^1^ Charité–Universitätsmedizin Berlin, corporate member of Freie Universität Berlin, Humboldt Universität zu Berlin, and Berlin Institute of Health, Stem Cell Facility, 10353 Berlin, Germany

^2^ Institute of Medical Immunology, Charité – Universitätsmedizin Berlin, corporate member of Freie Universität Berlin, Humboldt-Universität zu Berlin, and Berlin Institute of Health, Augustenburger Platz 1, Berlin, 13353 Germany

^3^ Department of Experimental Animal Facilities and Biorisk Management, Friedrich-Loeffler Institut, Greifswald-Insel Riems, Germany

^4^ Charité–Universitätsmedizin Berlin, corporate member of Freie Universität Berlin, Humboldt Universiät zu Berlin, and Berlin Institute of Health, Department of Pediatric Oncology and Hematology, 10353 Berlin, Germany

^5^ German Cancer Consortium (DKTK), 10117 Berlin, Germany

^6^ German Cancer Research Center (DKFZ), 69120 Heidelberg, Germany

^7^ Berlin Institute of Health at Charité - Universitätsmedizin Berlin, Charitéplatz 1, 10117 Berlin, Germany

^8^ Ardigen, 30-394 Kraków, Poland

^9^ Charité–Universitätsmedizin Berlin, corporate member of Freie Universität Berlin, Humboldt Universität zu Berlin, and Berlin Institute of Health, Department of Hematology, Oncology and Tumorimmunology, Charité – Universitätsmedizin Berlin, Berlin, Germany

Supplemental Table S1: Detailed summary of laboratory range results allow the comparisons between specimens’ samples (WB, mobilized WB before and after LA as well as cellular products) and cohorts (healthy donors with cohort 1: not-mobilized, cohort 2: G- CSF mobilized as well as patients cohort 3: G-CSF-mobilized).

| **Sample** | **Mobilization** | **Check point** | **Number** | **Parameter** | **results** | | | |
| --- | --- | --- | --- | --- | --- | --- | --- | --- |
|  |  |  |  |  | min | max | median |  |
| Donor  WB | yes | before apheresis | 15 | CD45 cells/µL | 28,860 | 84,620 | 56,280 |  |
|  |  |  |  | CD34 cells/µL | 32 | 280 | 120 |  |
|  |  |  |  | CD34 % | 0.11 | 0.47 | 0.19 |  |
|  |  |  |  | CD3 cells/µL | 1,536 | 4,736 | 3,228 |  |
|  |  |  |  | CD3 % | 3.17 | 9.78 | 5.48 |  |
|  |  |  |  | CD19 cells/µL | 382 | 1,772 | 750 |  |
|  |  |  |  | CD19 % | 0.90 | 2.30 | 1.31 |  |
| Donor  WB | no | n/a | 15 | CD45 cells/µL | 4,080 | 8,200 | 5,770 |  |
|  |  |  |  | CD34 cells/µL | 0 | 4 | 2 |  |
|  |  |  |  | CD34 % | 0 | 0.08 | 0.03 |  |
|  |  |  |  | CD3 cells/µL | 984 | 2,090 | 1,289 |  |
|  |  |  |  | CD3 % | 12.66 | 33.53 | 25.18 |  |
|  |  |  |  | CD19 cells/µL | 128 | 475 | 259 |  |
|  |  |  |  | CD19 % | 2.23 | 6.93 | 4.26 |  |
| Donor lymphocytes | no | LA | 20 | CD45 cells/µL | 29,730 | 114,090 | 78,315 |  |
|  |  |  |  | CD34 cells/µL | 8 | 267 | 57 |  |
|  |  |  |  | CD34 % | 0.01 | 0.24 | 0.07 |  |
|  |  |  |  | CD3 cells/µL | 16,112 | 67,272 | 41,666 |  |
|  |  |  |  | CD3 % | 27.81 | 62.72 | 48.86 |  |
|  |  |  |  | CD19 cells/µL | 1,784 | 16,120 | 8,373 |  |
|  |  |  |  | CD19 % | 4.27 | 17.22 | 10.04 |  |
| Allogenic hematopoietic stem cells | yes | LA | 30 | CD45 cells/µL | 116,695 | 691,240 | 280,805 |  |
|  |  |  |  | CD34 cells/µL | 460 | 4,800 | 2020 |  |
|  |  |  |  | CD34 % | 0.24 | 2.68 | 0.72 |  |
|  |  |  |  | CD3 cells/µL | 41,190 | 200,040 | 68,020 |  |
|  |  |  |  | CD3 % | 12.56 | 37.20 | 25.77 |  |
|  |  |  |  | CD19 cells/µL | 1,207 | 60,120 | 17,980 |  |
|  |  |  |  | CD19 % | 2.69 | 14.39 | 6.51 |  |
| Autologous hematopoietic stem cells | yes | LA | 30 | CD45 cells/µL | 64,490 | 783,840 | 166,390 |  |
|  |  |  |  | CD34 cells/µL | 111 | 35,440 | 1,285 |  |
|  |  |  |  | CD34 % | 0.13 | 11.48 | 1.12 |  |
|  |  |  |  | CD3 cells/µL | 2,977 | 70,440 | 23,662 |  |
|  |  |  |  | CD3 % | 3.63 | 43.29 | 14.84 |  |
|  |  |  |  | CD19 cells/µL | 0 | 6,670 | 266 |  |
|  |  |  |  | CD19 % | 0.00 | 3.03 | 0.19 |  |
