# Supplementary material for: Widely applicable, extended flow cytometric stem cell enumeration panel for quality control of advanced cellular products
Source: Sci Rep. 2022 Oct 26;12:17995. doi: 10.1038/s41598-022-22339-1 (PMC9605971; doi:10.1038/s41598-022-22339-1)
Supplement: Supplementary file 4 — Supplementary Table S2. [file 41598_2022_22339_MOESM4_ESM.docx]

**Widely applicable, extended flow cytometric stem cell enumeration panel for quality control of advanced cellular products**

Katy Haussmann^1,*^, Mathias Streitz^2,3^, Anna Takvorian^1^, Jana Grund^1^, Zemra Skenderi^1^, Carola Tietze-Bürger^1^, Kamran Movassaghi^1^, Annette Künkele^1,4-7^, Agnieszka Blum^8^, Lars Bullinger^1,5,6,9^

^1^ Charité–Universitätsmedizin Berlin, corporate member of Freie Universität Berlin, Humboldt Universität zu Berlin, and Berlin Institute of Health, Stem Cell Facility, 10353 Berlin, Germany

^2^ Institute of Medical Immunology, Charité – Universitätsmedizin Berlin, corporate member of Freie Universität Berlin, Humboldt-Universität zu Berlin, and Berlin Institute of Health, Augustenburger Platz 1, Berlin, 13353 Germany

^3^ Department of Experimental Animal Facilities and Biorisk Management, Friedrich-Loeffler Institut, Greifswald-Insel Riems, Germany

^4^ Charité–Universitätsmedizin Berlin, corporate member of Freie Universität Berlin, Humboldt Universiät zu Berlin, and Berlin Institute of Health, Department of Pediatric Oncology and Hematology, 10353 Berlin, Germany

^5^ German Cancer Consortium (DKTK), 10117 Berlin, Germany

^6^ German Cancer Research Center (DKFZ), 69120 Heidelberg, Germany

^7^ Berlin Institute of Health at Charité - Universitätsmedizin Berlin, Charitéplatz 1, 10117 Berlin, Germany

^8^ Ardigen, 30-394 Kraków, Poland

^9^ Charité–Universitätsmedizin Berlin, corporate member of Freie Universität Berlin, Humboldt Universität zu Berlin, and Berlin Institute of Health, Department of Hematology, Oncology and Tumorimmunology, Charité – Universitätsmedizin Berlin, Berlin, Germany

Supplemental Table S2: Detailed results of sensitivity measurements with the established approach and statistic evaluation of Limit of Detection (LOD) and Limit of Quantification (LOQ) with the help of Standard Deviation (SD) and the derivation of the Standard Deviation (SD’).

| **Statistic Values** | **Sample**  **Tube** | **Sensitivity**  **Established Approach** | | | | | |
| --- | --- | --- | --- | --- | --- | --- | --- |
|  |  | CD34 | | CD3 | | CD19 | |
|  |  | % | cells/µL | % | cells/µL | % | cells/µL |
|  | 1 | 0.05 | 3 | 0.18 | 3 | 0.01 | 2 |
|  | 2 | 0.04 | 2 | 0.32 | 4 | 0.06 | 6 |
|  | 3 | 0.03 | 2 | 0.51 | 7 | 0.03 | 3 |
|  | 4 | 0.01 | 1 | 0.25 | 3 | 0.02 | 2 |
|  | 5 | 0.02 | 1 | 0.44 | 6 | 0.05 | 4 |
|  | 6 | 0.03 | 2 | 0.51 | 7 | 0.06 | 6 |
|  | 7 | 0.04 | 3 | 0.44 | 6 | 0.05 | 4 |
|  | 8 | 0.02 | 1 | 0.30 | 4 | 0.06 | 6 |
|  | 9 | 0.02 | 1 | 0.17 | 2 | 0.05 | 4 |
|  | 10 | 0.04 | 2 | 0.36 | 5 | 0.04 | 3 |
| Standard Deviation (SD) |  | 0.01247219 | 0.78881064 | 0.12568037 | 1.7669811 | 0.01766981 | 1.56347192 |
| SD’ |  | 0.00394405 | 0.24944383 | 0.03974362 | 0.55876849 | 0.00558768 | 0.49441323 |
| 3*SD’ =LOD |  | 0.01183216 | 0.74833148 | 0.11923087 | 1.67630546 | 0.01676305 | 1.48323970 |
| **LOD** |  | **0.01** | **1** | **0.12** | **2** | **0.02** | **1** |
| 10*SD’ =LOQ |  | 0.03944053 | 2.49443826 | 0.39743623 | 5.58768487 | 0.05587685 | 4.94413232 |
| **LOQ** |  | **0.04** | **2** | **0.40** | **6** | **0.06** | **5** |
