# Supplementary material for: Widely applicable, extended flow cytometric stem cell enumeration panel for quality control of advanced cellular products
Source: Sci Rep. 2022 Oct 26;12:17995. doi: 10.1038/s41598-022-22339-1 (PMC9605971; doi:10.1038/s41598-022-22339-1)
Supplement: Supplementary file 6 — Supplementary Table S6. [file 41598_2022_22339_MOESM6_ESM.docx]

**Widely applicable, extended flow cytometric stem cell enumeration panel for quality control of advanced cellular products**

Katy Haussmann^1,*^, Mathias Streitz^2,3^, Anna Takvorian^1^, Jana Grund^1^, Zemra Skenderi^1^, Carola Tietze-Bürger^1^, Kamran Movassaghi^1^, Annette Künkele^1,4-7^, Agnieszka Blum^8^, Lars Bullinger^1,5,6,9^

^1^ Charité–Universitätsmedizin Berlin, corporate member of Freie Universität Berlin, Humboldt Universität zu Berlin, and Berlin Institute of Health, Stem Cell Facility, 10353 Berlin, Germany

^2^ Institute of Medical Immunology, Charité – Universitätsmedizin Berlin, corporate member of Freie Universität Berlin, Humboldt-Universität zu Berlin, and Berlin Institute of Health, Augustenburger Platz 1, Berlin, 13353 Germany

^3^ Department of Experimental Animal Facilities and Biorisk Management, Friedrich-Loeffler Institut, Greifswald-Insel Riems, Germany

^4^ Charité–Universitätsmedizin Berlin, corporate member of Freie Universität Berlin, Humboldt Universiät zu Berlin, and Berlin Institute of Health, Department of Pediatric Oncology and Hematology, 10353 Berlin, Germany

^5^ German Cancer Consortium (DKTK), 10117 Berlin, Germany

^6^ German Cancer Research Center (DKFZ), 69120 Heidelberg, Germany

^7^ Berlin Institute of Health at Charité - Universitätsmedizin Berlin, Charitéplatz 1, 10117 Berlin, Germany

^8^ Ardigen, 30-394 Kraków, Poland

^9^ Charité–Universitätsmedizin Berlin, corporate member of Freie Universität Berlin, Humboldt Universität zu Berlin, and Berlin Institute of Health, Department of Hematology, Oncology and Tumorimmunology, Charité – Universitätsmedizin Berlin, Berlin, Germany

Supplemental Table S6: Repeatability results after adding respective antibodies to the pre-formulated reagent panel including CD45 FITC, CD34 PE, CD3 PB, CD19 APC, 7-AAD as well as CD16 PC7, CD56-PC7 and counting beads.

| **Parameter** | **Sample Tube** | | | | | | **Statistic Values** | | |
| --- | --- | --- | --- | --- | --- | --- | --- | --- | --- |
|  | **1** | **2** | **3** | **4** | **5** | **mean** | | **SD** | **CV** |
| CD45 cells/µL | 7279 | 7483 | 7257 | 7285 | 7406 | 7342 | | 98.0051 | 1.33 |
| CD34 cells/µL | 2 | 1 | 2 | 2 | 1 | 2 | | 0.5477 | 34.23 |
| CD3 cells/µL | 1910 | 1976 | 1897 | 1886 | 1923 | 1918 | | 35.0614 | 1.83 |
| CD19 cells/µL | 305 | 304 | 295 | 303 | 302 | 302 | | 3.9623 | 1.31 |
| CD3-/CD16/CD56 cells/µL | 226 | 236 | 237 | 228 | 235 | 232 | | 5.0299 | 2.16 |
| CD34 cells % | 0,03 | 0.02 | 0.02 | 0.03 | 0.01 | 0.02 | | 0.0084 | 38.03 |
| CD3 cells % | 26.25 | 26.41 | 26.14 | 25.89 | 25.97 | 26.13 | | 0.2098 | 0.80 |
| CD19 cells % | 4.19 | 4.06 | 4.06 | 4.16 | 4.08 | 4.11 | | 0.0608 | 1.48 |
| CD3-/CD16/CD56 cells % | 9.03 | 9.13 | 9.48 | 9.19 | 9.30 | 9.23 | | 0,1724 | 1,87 |
