# Supplementary material for: Widely applicable, extended flow cytometric stem cell enumeration panel for quality control of advanced cellular products
Source: Sci Rep. 2022 Oct 26;12:17995. doi: 10.1038/s41598-022-22339-1 (PMC9605971; doi:10.1038/s41598-022-22339-1)
Supplement: Supplementary file 7 — Supplementary Table S7. [file 41598_2022_22339_MOESM7_ESM.docx]

**Widely applicable, extended flow cytometric stem cell enumeration panel for quality control of advanced cellular products**

Katy Haussmann^1,*^, Mathias Streitz^2,3^, Anna Takvorian^1^, Jana Grund^1^, Zemra Skenderi^1^, Carola Tietze-Bürger^1^, Kamran Movassaghi^1^, Annette Künkele^1,4-7^, Agnieszka Blum^8^, Lars Bullinger^1,5,6,9^

^1^ Charité–Universitätsmedizin Berlin, corporate member of Freie Universität Berlin, Humboldt Universität zu Berlin, and Berlin Institute of Health, Stem Cell Facility, 10353 Berlin, Germany

^2^ Institute of Medical Immunology, Charité – Universitätsmedizin Berlin, corporate member of Freie Universität Berlin, Humboldt-Universität zu Berlin, and Berlin Institute of Health, Augustenburger Platz 1, Berlin, 13353 Germany

^3^ Department of Experimental Animal Facilities and Biorisk Management, Friedrich-Loeffler Institut, Greifswald-Insel Riems, Germany

^4^ Charité–Universitätsmedizin Berlin, corporate member of Freie Universität Berlin, Humboldt Universiät zu Berlin, and Berlin Institute of Health, Department of Pediatric Oncology and Hematology, 10353 Berlin, Germany

^5^ German Cancer Consortium (DKTK), 10117 Berlin, Germany

^6^ German Cancer Research Center (DKFZ), 69120 Heidelberg, Germany

^7^ Berlin Institute of Health at Charité - Universitätsmedizin Berlin, Charitéplatz 1, 10117 Berlin, Germany

^8^ Ardigen, 30-394 Kraków, Poland

^9^ Charité–Universitätsmedizin Berlin, corporate member of Freie Universität Berlin, Humboldt Universität zu Berlin, and Berlin Institute of Health, Department of Hematology, Oncology and Tumorimmunology, Charité – Universitätsmedizin Berlin, Berlin, Germany

Supplemental Table S7: Sensitivity measurement of the established approach after adding respective antibodies to the pre-formulated reagent panel including CD45 FITC, CD34 PE, CD3 PB, CD19 APC, 7-AAD as well as CD16 PC7, CD56-PC7 and counting beads to determine the Limit of Detection (LOD) and Limit of Quantification (LOQ) for CD3-/CD16/CD56 natural killer cells.

| **Statistic Values** | **Sample**  **Tube** | **Sensitivity**  **Established Approach** |
| --- | --- | --- |
|  |  | CD3-/CD16/CD56  cells/µL |
|  | 1 | 2 |
|  | 2 | 2 |
|  | 3 | 2 |
|  | 4 | 2 |
|  | 5 | 2 |
|  | 6 | 2 |
|  | 7 | 1 |
|  | 8 | 1 |
|  | 9 | 3 |
|  | 10 | 2 |
| Standard Deviation (SD) |  | 0,567646212 |
| SD’ |  | 0,179505494 |
| 3*SD’ =LOD |  | 0,538516481 |
| **LOD** |  | **1** |
| 10*SD’ =LOQ |  | 1,795054936 |
| **LOQ** |  | **2** |
